# Computational Study on Selective PDE9 Inhibitors on PDE9-Mg/Mg, PDE9-Zn/Mg, and PDE9-Zn/Zn Systems

**DOI:** 10.3390/biom11050709

**Published:** 2021-05-10

**Authors:** Dakshinamurthy Sivakumar, Sathishkumar Mudedla, Seonghun Jang, Hyunjun Kim, Hyunjin Park, Yonwon Choi, Joongyo Oh, Sangwook Wu

**Affiliations:** 1 R&D Center, Pharmcadd, 221, 17 APEC-ro, Haeundae-gu, Busan 48060, Korea; sivabioinfo@gmail.com (D.S.); mudedla@pharmcadd.com (S.M.); jwjang@pharmcadd.com (S.J.); 2 R&D Center, Huons co. Ltd., Ansan-si 15588, Korea; hjkim0206@huons.com (H.K.); phj0630@huons.com (H.P.); cyw@huons.com (Y.C.); ohjg69@huons.com (J.O.)

**Keywords:** selective inhibitors, PDE9, molecular dynamics, metal complexes, BAY73-6691

## Abstract

PDE9 inhibitors have been studied to validate their potential to treat diabetes, neurodegenerative disorders, cardiovascular diseases, and erectile dysfunction. In this report, we have selected highly potent previously reported selective PDE9 inhibitors BAY73-6691R, BAY73-6691S, 28r, 28s, 3r, 3s, PF-0447943, PF-4181366, and 4r to elucidate the differences in their interaction patterns in the presence of different metal systems such as Zn/Mg, Mg/Mg, and Zn/Zn. The initial complexes were generated by molecular docking followed by molecular dynamics simulation for 100 ns in triplicate for each system to understand the interactions’ stability. The results were carefully analyzed, focusing on the ligands’ non-bonded interactions with PDE9 in different metal systems.

## 1. Introduction

Cellular functions are regulated by the integration of intra and extracellular signals. Several signal transduction systems evolved to receive and process the signals; dysregulations lead to disruption in cellular functions and leads to diseases in humans [1]. One of the earliest identified cyclic nucleotide signalling systems is cAMP and cGMP. Phosphodiesterases (PDEs), a superfamily of enzymes, specifically hydrolyzes cyclic AMP and cyclic GMP, are detected in various tissues such as lungs, kidney, heart, brain, prostate, small intestine, prostate, bladder, and hematopoietic cells [2,3,4,5]. PDE isoenzymes differing by their substrate specificity are classified into 11 families, which encode several isoforms. PDEs- (5,6,9) are cyclic GMP substrate-specific, whereas PDEs- (4,7,8) are specific to cyclic AMP [6,7].

PDEs were initially identified as the key regulators of intracellular cyclic nucleotide concentrations and later confirmed the role as host involving these secondary messengers in health and disease [8,9,10]. Each PDE holds its critical positions in regulating specific cyclic nucleotide signalling pathways and pathophysiological responses. Targeting particular PDEs by selective PDE inhibitors can improve the treatment efficacy. Some of the ongoing PDE targeting such as metabolic and cardiovascular diseases with PDE5 inhibitors, vascular remodelling with PDE1C and PDE3A inhibitors, and schizophrenia with PDE1 and PDE3A inhibitors, PDE7 inhibitors for inflammation, diabetes with PDE4 inhibitors and a combination of the above depend on the disease conditions [1].

**Figure 1:**
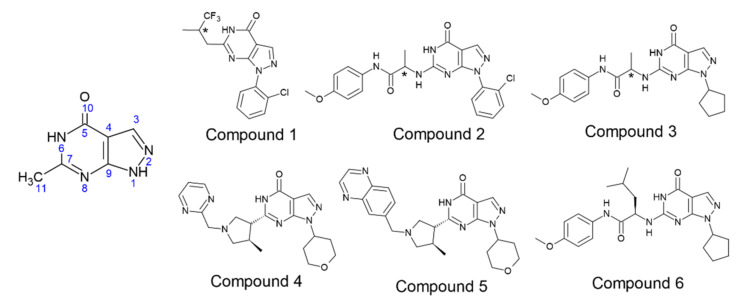
Selected literature reported chemical structures of PDE9 inhibitors [18]. Compound **1** (BAY73-6691), Compound **2** (28r and 28s), Compound **3** (3r and 3s), Compound **4** (PF-04447943), Compound **5** (PF-4181366), and Compound **6** (4r). The chiral carbon that makes two enantiomers were marked with symbol (*). Common substructure included separately with numbering.

In this work, we focused on PDE9 and several potent PDE9 inhibitors, which are already patented for the treatment of Diabetes mellitus, Alzheimer’s disease and have shown potential for the treatment of obesity and cardiovascular diseases [11,12]. Although PDE9 inhibitors hold several advantages and sound therapeutic effects, minimal research is carried out compared to other PDE families. Here, we are analyzing the literature reported, highly potent PDE9 inhibitors BAY73-6691, PF-04447943, PF-4181366, 28 (r and s), 3 (r and s), and 4r (Figure 1) [13,14,15,16,17].

BAY73-6691 identified as a selective and potent PDE-9 inhibitor that can be used to treat neurodegenerative disorders such as Alzheimer’s disease, as seen from the improved learning and memory in rats [18]. Additionally, in sickle cell disease, BAY73-6691 reduces neutrophil adhesion, inhibiting the vaso-occlusive process [4]. BAY73-6691 can block the PDE9 activity in the corpus cavernosum, which amplifies the Nitric Oxide -cGMP-mediated cavernosal responses, resulting in improvement in erectile dysfunction treatment [19]. PF-04447943 was identified as a potent and selective inhibitor of the PDE9A completed phase- Ib trials in Sickle disease patients to study the safety, PK/PD, and tolerability [20,21,22]. The study carried out in rats and mice showed improvement in cognition function and is currently in the clinical development for Alzheimer’s disease [21].

Compound **3**(3r) shows that it can act as a potential hypoglycemic agent by inhibiting mRNA expression of phosphoenolpyruvate carboxykinase (PEPCK) and Fructose 1,6-bisphosphatase (F-6-Pase) [17]. The other compounds show the effective inhibitory activity of PDE-9, as reported elsewhere [17]. Compound **5** (PF-4181366) reported a selective and highly potent inhibitor for PDE9A and it was identified that it could cause a dose-dependent increase in cGMP levels [15]. Compound **5** increases cGMP level in the striatum, hippocampus, and cortex with variations [23]. The rest of the compounds were also identified as PDE-9 inhibitors and were considered for the study.

## 2. Materials and Methods

### 2.1. Protein Preparation

The protein structure of Phosphodiesterase 9A was downloaded from the protein databank with Pdb id:4QGE [24]. PDE9 (Zn/Mg) was used from the above system, PDE9(Mg/Mg) and PDE9(Zn/Zn) developed by replacing Zn with Mg. Protein structures were prepared by correcting the bond orders, adding missing hydrogens, optimizing H-bond with protonation states of residues at pH 7.0, and restraining minimization for added hydrogens using OPLS2005 forcefield by protein preparation wizard [25]. We have performed DFT calculations for the ligands to minimize the geometry to get equilibrium bond lengths, bond angles and torsional angles for MD simulation. The charges derived using DFT methods are of high quality compared to other existing autogenerated programs based on low-level semiempirical methods.

### 2.2. Ligand Topology

PDE9 inhibitors used in the study were initially optimized using the DFT method with Gaussian 16 [26] on B3LYP functionals [27] and 6-31G (d,p) basis set to calculate the electrostatic potential. The antechamber was run for the Gaussian output file to get GAFF atom types with RESP charges [28]. Atom types and all the needed ligand parameters were obtained from the above process.

### 2.3. Molecular Docking

Docking with explicit waters was performed with the Autodock4 Hydrated Docking method [29]. We used the crystal structure of 4QGE for explicit water position. Three water molecules were obtained, one interacting with the backbone of I403, N405, and E406, another interacting only with N405, and the other interacting with D402 and H256. All of those water molecules were positioned near the pyrazolo-pyrimidine moiety, typical for all docked compounds. We considered those as dummy atoms during the autodock hydrated docking procedure, as described in detail. After that, we removed the remaining water molecules and modified their positions to 4QGE. For other compounds, we aligned to the cocrystal structure of 4QGE and repeated the same procedure as described above for the dummy water atom. The binding grid was set, keeping the active site as the centre.

### 2.4. Molecular Dynamics Simulations

The stability of the complexes was studied by MD simulations using Gromacs2019 [30]. The Amber99sb-ildn force field was used for the protein parameters [31]. The protein-ligand complexes were solvated explicitly using the TIP3P water model inside the cubic box, and its size extends 0.1 nm away from the protein on the edges of the box in each direction. The system’s overall charge was neutralized by adding a 0.1 M salt concentration (Na^+^Cl^−^). All the simulation was carried out in the GPU enabled Linux clusters. The entire system was minimized until the maximum force was less than 10 kj/mol with the maximum steps of 50,000. The system was then equilibrated for 5 ns under NVT conditions with temperature coupling for two separate groups protein-ligand and water-ions, at 300 K. The bonds to the hydrogen atoms were constrained using the Lincs algorithm [32]. The temperature and pressure were kept constant by the Berendsen thermostat and V-rescale, respectively [33,34]. The long-range electrostatic interactions were calculated using the particle mesh Ewald method (PME) [35]. The cutoff distances for Coulomb and van der Waals interactions were set as 1.2 nm. The final production run was carried out for 100 ns in triplicate (3 × 100 ns) at a temperature of 300 K and a pressure of 1 bar. More detailed methodology and analysis can be seen in our previous reports [36,37].

### 2.5. MM/PBSA Binding Free Energy Calculation

The g_mmpbsa package was used to calculate molecular mechanics (MM)/Poisson-Boltzmann surface area (PBSA) free energy calculation [38]. The last 20 ns from the final production run were used for the analysis of the binding free energy. The calculated binding free energy contains three energetic terms, polar solvation energy (PSA), nonpolar solvation energy, and potential energy in a vacuum. Bonded (angle, bond, and dihedral) and non-bonded (electrostatic and van der Waal) interactions included in the vacuum’s potential energy were calculated using mm forcefield parameters. The other two energetic terms, polar solvation energy and nonpolar solvation energy, were calculated using the Poisson-Boltzmann equation and the solvent-accessible surface area (SASA) model.

The binding free energy calculated by the following equation,

∆G_binding_ = ∆G_complex_ − (∆G_protein_ + ∆G_ligand_) (1)

∆Gcomplex, ∆Gprotein, and ∆Gligand is the total free energy of the protein-ligand complex, isolated protein, and isolated ligand, respectively.

## 3. Results and Discussion

### 3.1. Docking of PDE-9 Inhibitors with PDE-9

The PDE-9 structure docked with selected inhibitors to generate the complex. Compound **1** (both *R* and *S*) holds the same dock score of −9.5 kcal/mol and the identical interacting residues, hydrogen bond interactions with Gln453, and hydrophobic interactions with Phe251 and Phe456. Compound 2R with the docking score of −11.3 kcal/mol shows hydrogen bond interactions with Ala452 and Gln453, hydrophobic interactions with Phe251 and Phe456. Compound **2** (*S*) with a docking score of −10.9 kcal/mol shows strong hydrogen bond interactions with Gln453 (with N6 and O10 atom of the pyrimidine ring) and Ala452 (with N11 atom) and signature π-π interactions formed between Phe456 and both pyrimidine-pyrazole rings of the Compound 2S. Compound 3R with the docking score of −10.7 kcal/mol shows π-π interactions with Phe251 and Phe256, hydrogen bond interactions with Gln453 and Tyr424. However, Compound 3S, though it has a similar dock score (−10.3 kcal/mol), has a different interacting pattern. Hydrogen bond interactions with Gln453 and Ala452 and hydrophobic interactions with Phe456. Compound **4** with a docking score of −10.7 kcal/mol shows hydrogen bond interactions with Gln453 and hydrophobic interactions with Phe251 and Phe456. Compound **5** with the highest dock score of −12.0 kcal/mol and hydrogen bond interaction with Gln453 and hydrophobic interactions with Phe251 and Ph3456. Compound **6** with a docking score of −9.3 kcal/mol has hydrogen bond interactions with Ala452 and Gln453, also hydrophobic interactions with Phe456 (Table 1).

### 3.2. Interaction Details Based on the Simulation Results

#### 3.2.1. Compound **1** (BAY73-6691 -*R* and *S*) in PDE-9 Zn/Mg, PDE-9 Mg/Mg, and PDE-9 Zn/Zn

**Figure 2:**
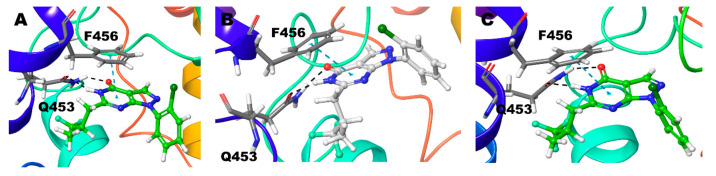
Non-bonded interactions of Compound **1**(*R*) in (**A**) PDE9 Zn/Mg (**B**) PDE9 Mg/Mg (**C**) PDE9 Zn/Zn.

**Figure 3:**
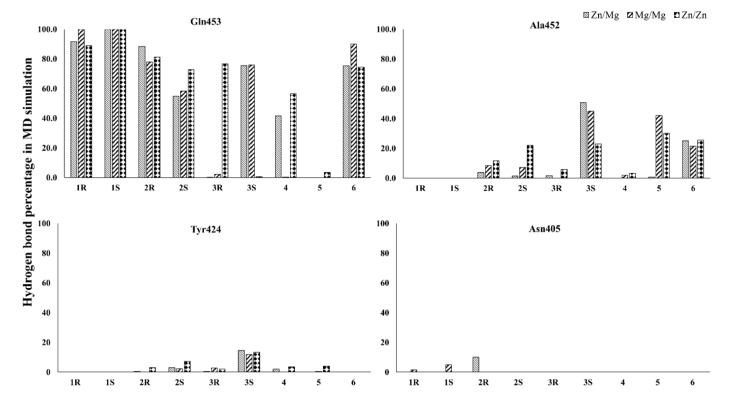
The strength of the hydrogen bond interactions between the critical residues of PDE-9 (in Zn/Mg, Mg/Mg, and Zn/Zn) and the compounds was monitored throughout the simulations.

Compound **1** (BAY73-6691 (*R*)) retains the native interaction in both PDE-9 Zn/Mg, Mg/Mg, and Zn/Zn models. The O and adjacent N atom of the pyrimidine ring form hydrogen bond interactions with the Gln453, and the pyrazolo-pyrimidine ring forms π-π stacking with Phe456 (Figure 2) (Table 2). Gln453 forms the hydrogen bond interactions for more than 90% of the simulation time in all three cases (Figure 3).

**Figure 4:**
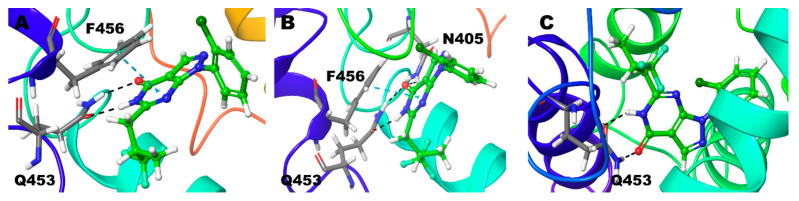
Non-bonded interactions of Compound **1**(*S*) in (**A**) PDE9 Zn/Mg (**B**) PDE9 Mg/Mg (**C**) PDE9 Zn/Zn.

Compound **1** (BAY73-6691 (*S*)) in the Zn/Mg model follows similar interactions as above; in the Mg/Mg model similar interaction and additional hydrogen bond interaction occurs towards Asn405. Still, Zn/Zn model holds the same hydrogen bond interaction as the Zn/Mg model, but the signature π-π stacking with Phe456 was missing (Figure 4) (Table 2). The S-conformer of the Compound **1** forms a hydrogen bond with active site residue Gln453 for whole (100%) simulation in all the cases and additionally 5% of the simulation time with Asn405 in Mg/Mg case (Figure 3).

#### 3.2.2. Compound **2** (28*R* and 28*S*) in PDE-9 Zn/Mg, PDE-9 Mg/Mg, and PDE-9 Zn/Zn

**Figure 5:**
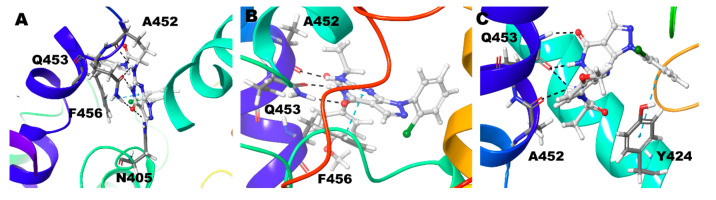
Non-bonded interactions of Compound **2** (*R*) in (**A**) PDE9 Zn/Mg (**B**) PDE9 Mg/Mg (**C**) PDE9 Zn/Zn.

In the 28R-PDE-Zn/Mg complex, the N6 atom of the pyrimidine ring shows strong hydrogen bond interaction only with Gln453. N11atom, linked with the C7 atom of the pyrimidine ring, shows hydrogen bond interactions with both Gln453 and Ala452. Likewise, the O10 atom (attached with the C5 atom of the pyrimidine ring) shows strong hydrogen bond interaction with both Gln453 and Asn405. The signature π-π stacking of the pyrimidine ring and Phe456 was maintained in the simulation (Figure 5A) (Table 2). Gln453 forms hydrogen bond for 88% of the simulation time and Asn405 for 10% of the simulation time, and Ala452 forms 4% of the simulation time (Figure 3).

In Compound 2R-PDE-Mg/Mg complex, though similar interactions were observed as in the Zn/Mg complex, it seems as if the intensity of the interactions is reduced. N6 atom of the pyrimidine ring, along with the O10 shows hydrogen bond interaction with the Gln453. In this O10 atom interaction, it is seen only with Gln453 and not with Asn405, as seen in Zn/Mg system. Similarly, the N11 atom shows hydrogen bond interaction only with Ala452 and not with Gln453, as we observed in the Zn/Mg system. The signature π-π stacking of the pyrimidine ring and Phe456 was maintained in this simulation (Figure 5B) (Table 2). Gln453 and Ala452 form hydrogen bonds with the compound for 78% and 8% of the simulation time (Figure 3).

In Compound 2R-PDE-Zn/Zn complex, very similar interactions were observed as in the Mg/Mg complex with only additional π-π interaction of Tyr424 with phenyl ring substituted the N1 atom of the pyrazole (Figure 5C) (Table 2). In nearly 81% of the simulation time Gln453 forms hydrogen bond, 12% of the time with Ala452, and less than 3% with Tyr424 (Figure 3).

**Figure 6:**
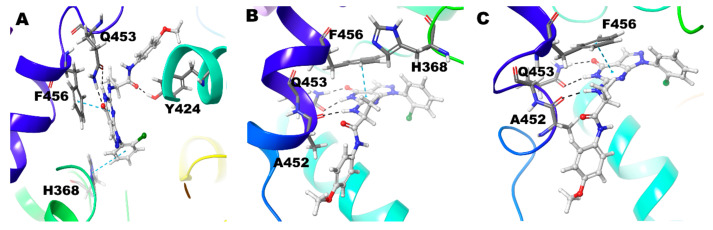
Non-bonded interactions of Compound **2**(*S*) in (**A**) PDE9 Zn/Mg (**B**) PDE9 Mg/Mg (**C**) PDE9 Zn/Zn.

In Compound 2S, PDE-Zn/Mg, and Mg/Mg complex, the N6 atom of the pyrimidine ring and the O10 show hydrogen bond interaction with the Gln453 (Figure 6A). The compound also forms additional hydrogen bond interaction with Tyr424 in Zn/Mg model and with Ala452 in Mg/Mg model (Figure 6B). π-π stacking was observed between pyrimidine ring, phenyl ring substituted at N1 atom of the pyrazole with Phe456 and His 368, respectively. In the Zn/Zn model, there are similar interactions to the above cases, except no hydrophobic interaction was observed with His368 (Figure 6C) (Table 2). Gln453 shows interactions around 55% of the simulation time in the Zn/Mg and Mg/Mg case, whereas, in Zn/Zn, the interaction lasts more than 73% of the simulation time. Similarly, Tyr424 shows 2 to 3% of the simulation time in the first two cases, but in Zn/Zn, it lasts 7%. In the case of Ala452, short-lived interaction occurred with just 1.5% in Zn/Mg and 7% in Mg/Mg, but 22% simulation time in Zn/Zn (Figure 3).

#### 3.2.3. Compound 3 (3R and 3S) in PDE-9 Zn/Mg, PDE-9 Mg/Mg, and PDE-9 Zn/Zn

**Figure 7:**
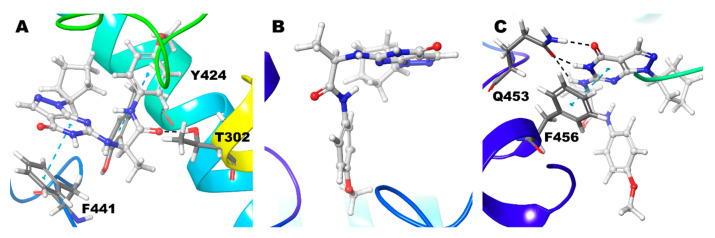
Non-bonded interactions of Compound **3**(*R*) in (**A**) PDE9 Zn/Mg (**B**) PDE9 Mg/Mg (**C**) PDE9 Zn/Zn.

Compound **3** (3*R*), Zn/Mg shows no hydrogen bond interactions from its pyrimidine-pyrazole moiety and no interactions with the active site residue Gln453. The pyrimidine ring forms π-π interaction with other phenylalanine (Phe441), not the usual Phe456. Other ligand parts show hydrogen bond interaction with Thr302 and a π-π interaction with Tyr424 (Figure 7A) (Table 2).

Interestingly, the Compound **3** (3*R*)–Mg/Mg model has no interactions observed (Figure 7B). In Zn/Zn model, the Gln453 shows strong hydrogen bond interactions with 3 different atoms (O10, N6, and N11) of the ligand. For more than 75% of the simulation time, the interactions were maintained (Figure 3). In this case, Signature π-π stacking was observed between the pyrimidine ring and Phe456 (Figure 7C) (Table 2). 

**Figure 8:**
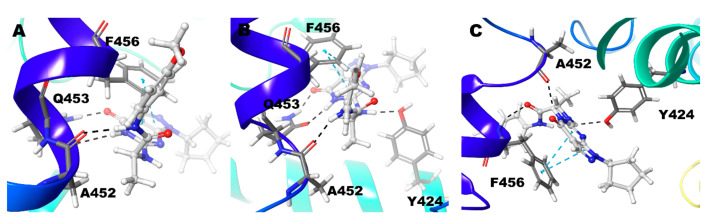
Non-bonded interactions of Compound **3** (*S*) in (**A**) PDE9 Zn/Mg (**B**) PDE9 Mg/Mg (**C**) PDE9 Zn/Zn.

Compound **3** (3S), Zn/Mg and Mg/Mg model, strong hydrogen bond interaction N6 and O10 atom of the ligand with Gln453. Signature π-π stacking was observed between both the pyrimidine, pyrazole ring and Phe456. Ala452 also shows hydrogen bond interactions with the ligand for 50% and 45% of the simulation time, respectively (Figure 3). Additionally, in Mg/Mg model, Tyr424 shows hydrogen bond interaction with the N11 atom of ligand (Figure 8A,B). However, in the Zn/Zn model, the results are quite different from the other two models. Gln453 hydrogen bond is missing; N6 atom of the pyrimidine ring forms hydrogen bond interaction with the Ala452 for nearly 25% of the simulation time (Figure 3). The typical interaction with the previous two models is the signature π-π stacking of Phe456 with both the pyrimidine and pyrazole ring. The same amino acid also adds additional hydrogen bond interactions with the ligand. As we have seen in the Mg/Mg model, we can see that the N11 atom of the ligand shows hydrogen bond interaction with the Tyr424 (Figure 8C).

In contrast to Compound 3R, in which interaction with Gln453 of the Zn/Zn model show more than 75% of the simulation time, in this case, its vice versa; Zn/Zn has only negligible interactions. In contrast, the other two cases have interactions with the compound for more than 75% of the simulation time with Gln453. Tyr424 holds interactions with Compound 3S in all three instances for around 10 to 15% of the simulation time (Figure 3) (Table 2).

#### 3.2.4. Compound 4 (PF-04447943) in PDE-9 Zn/Mg, PDE-9 Mg/Mg, and PDE-9 Zn/Zn

**Figure 9:**
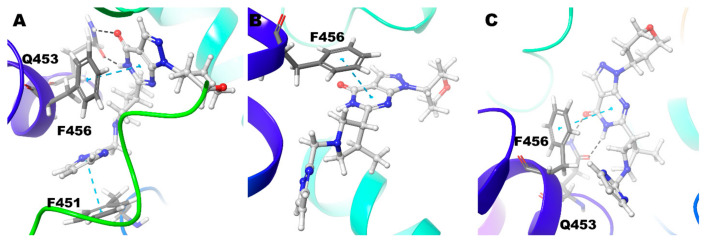
Non-bonded interactions of Compound **4** in (**A**) PDE9 Zn/Mg (**B**) PDE9 Mg/Mg (**C**) PDE9 Zn/Zn.

Compound **4** with Zn/Mg and Zn/Zn model holds signature interactions, hydrogen bond interactions with Gln453, and π-π stacking with Phe456 (Figure 9). Additionally, Phe441 shows π-π stacking with the ligand only in the Zn/Mg model. In the Mg/Mg model, the ligand shows no hydrogen bond interactions but holds only one π-π stacking between Phe456 and pyrimidine ring of the ligand (Figure 9) (Table 2). Hydrogen bond interaction with Gln453 for nearly 40% and 60% of the simulation time occurs only in Zn/Mg and Zn/Zn, respectively (Figure 3). Tyr424 and Ala452, in all cases, hold interactions only for a little time. 

#### 3.2.5. Compound **5** (PF-4181366) in PDE-9 Zn/Mg, PDE-9 Mg/Mg and PDE-9 Zn/Zn

**Figure 10:**
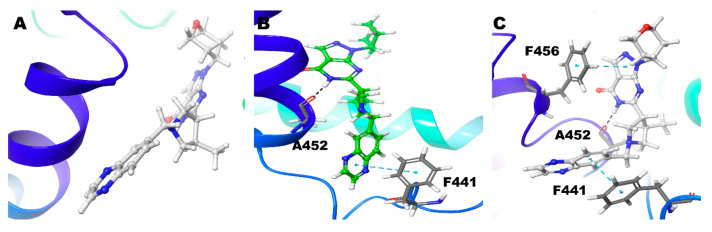
Non-bonded interactions of Compound **5** in (**A**) PDE9 Zn/Mg (**B**) PDE9 Mg/Mg (**C**) PDE9 Zn/Zn.

Compound **5** in the Zn/Mg model, with no significant interactions found (Figure 10A). Still, in Mg/Mg, though no signature interactions were found, Ala452 show hydrogen bond interactions with N6 atom and Phe441 showed π-π interactions with the ligand (Figure 10B). Additionally, in the Zn/Zn model, the N6 atom showed hydrogen bond interaction with Ala452; two π-π interaction were observed, one with Phe441 and another one signature π-π interaction–pyrazole ring with Phe456 (Figure 10C) (Table 2). Hydrogen bond interactions with Ala452 in Mg/Mg hold 42% and only 30% of the simulation time in the Zn/Zn model (Figure 3). Other interactions were not observed for a significant duration.

#### 3.2.6. Compound **6** (4r) in PDE-9 Zn/Mg, PDE-9 Mg/Mg and PDE-9 Zn/Zn

**Figure 11:**
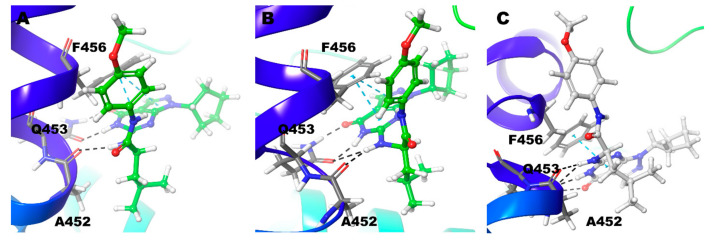
Non-bonded interactions of Compound **6** in (**A**) PDE9 Zn/Mg (**B**) PDE9 Mg/Mg (**C**) PDE9 Zn/Zn.

In the Zn/Mg model, hydrogen bond interaction was found between N6 and O10 atom of the ligand and Gln453 for nearly 75% of the simulation time. N11 atom showed hydrogen bond interactions with Ala452 for almost one-fourth of the simulation time. Phe456 showed π-π interactions with pyrimidine and pyrazole rings of Compound **6** (Figure 11A and Figure 3). It is very similar to the Mg/Mg model but only with an additional hydrogen bond for Gln453 with the N11 atom of the ligand (Figure 11B) (Table 2). In the Mg/Mg case, Gln453 holds interaction for 90% of the simulation time, whereas with Ala452 only around 20% of the simulation time (Figure 3). In the Zn/Zn model, similar to Zn/Mg interaction with a tiny difference, Phe456 shows π-π interactions only with the ligand’s pyrimidine ring (Figure 11C). Gln453 holds nearly 75% of the simulation as in the case of Zn/Mg. Ala452 also has, similar to Zn/Mg, 25% of the simulation time (Figure 3).

**Figure 12:**
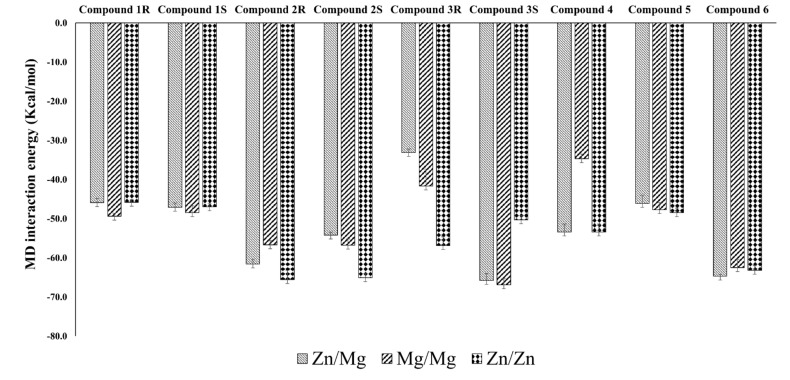
MD interaction energy was calculated for the compounds in PDE-9 (in Zn/Mg, Mg/Mg, and Zn/Zn).

Based on the molecular dynamics interaction energy (Figure 12), Compound **6** seems more effective in binding with PDE9 irrespective of the metal system. Compound 2R is also almost on par with Compound **6** in terms of binding, except for a bit less in the Mg/Mg system. Compound 3S shows better binding on par with the above two compounds except for in the Zn/Zn system. Compound 1R and 1S have a lesser binding affinity compared to other compounds in all the systems. Some compounds show better binding in some metal systems and not in others such as Compound 3R, which has higher preferences for Zn/Zn than Zn/Mg, and in Compound **4**, which is not so favourable with thw Mg/Mg system compared to the other two systems. The RMSD and RMSF plots obtained from the molecular dynamics results were shown in the Supplementary file (Figures S1–S18).

**Figure 13:**
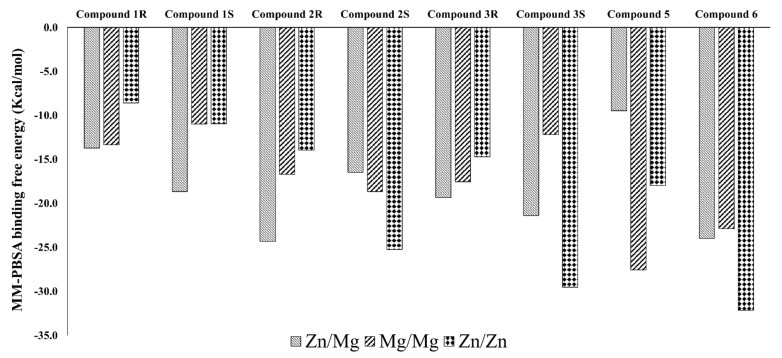
MM-PBSA binding free energy was calculated for the compounds in different metal systems.

Even the MM-PBSA based binding free energy analysis (Figure 13) also shows that Compound **6** is preferable in all three metal complexes. As seen in the MD interaction energy results, we have also noticed Compounds 1*R* and S least preference related to the Compound **6**. Inclination towards the different metal complexes differs among the compounds, as Compound **6**, 3S, and 2S are more inclined towards the Zn/Zn metal systems. Compound **5** is more inclined towards the Mg/Mg metal system, and Compounds 2R and 1S are relatively higher in preference over the Zn/Mg metal system than the other metal systems in PDE-9 inhibition. Compound **4** from the MM-PBSA results was omitted due to failure in the calculations due to unknown reasons after several attempts. The electrostatic interaction calculated using APBS calculation for Compound **3**S and Compound **6** for three different metal systems (Figures S19 and S20) did not show much significant differences in the binding region.

## 4. Conclusions

Overall, Compounds **1***R* and *S*, **2***R*, and Compound **6** show hydrogen bond interactions with active site residue Gln453 for more than 75% of the simulation time irrespective of the metal systems. Interestingly, 3R shows only strong interactions with the Zn/Zn system, whereas the other enantiomer of the same Compound 3S show interactions except for Zn/Zn. Compound **2** shows significant interaction in all three cases but with a slightly higher preference over the Zn/Zn system. Considering the interaction with Ala452, Compounds 3S and **6** show relatively equal interactions in all three systems. Compound **5** also extends its interactions with Ala452, with the execption of in Zn/Mg. Ty424 and Asn405 interactions are less significant in terms of our MD results. Based on this result, we can see that Compound **6** shows a stronger preference to PDE-9 irrespective of the metal systems, which is also well aligned with our other analysis based on the MD interaction energy and MM-PBSA binding free energy studies. The purpose of this study is not to claim one of the compounds as best, as all the compounds have shown efficacy and selectivity, and some are in different phases of clinical trials. Here we show the significance of the metal system of the PDE-9 and its influence in altering the inhibitory effect of the protein. Further analysis at the QM level and experimental validations to understand the impact of the different metal systems, including Mn/Mn, in the PDE-9 with ligand binding are under progress in our lab.

## Figures and Tables

**Table 1:** Molecular docking and interacting residues along with the score for all the complexes and available enantiomers.

Compound ID	Enantiomer	Docking Score (kcal/mol)	Interacting Residues *
Compound **1**	R	−9.5	F251, **Q453**, F456
Compound **1**	S	−9.5	F251, **Q453**, F456
Compound **2**	R	−11.3	F251, **A452**, **Q453**, F456
Compound **2**	S	−10.9	**A452**, **Q453**, F456
Compound **3**	R	−10.7	F251, **Y424**, **Q453**, F456
Compound **3**	S	−10.3	**A452**, **Q453**, F456
Compound **4**	n/a	−10.7	F251, **Q453**, F456
Compound **5**	n/a	−12.0	F251, **Q453**, F456
Compound **6**	n/a	−9.3	**A452**, **Q453**, F456

* Hydrogen bond residues are highlighted in bold.

**Table 2:** Inter-molecular interactions observed in the molecular dynamics studied in different systems.

Compound ID/Enantiomer	Metal System	Hydrogen Bond Interactions	Hydrophobic Contacts
Compound **1** (*R*)	Zn/Mg	Q453	F456
	Mg/Mg	Q453	F456
	Zn/Zn	Q453	F456
Compound **1** (*S*)	Zn/Mg	Q453	F456
	Mg/Mg	Q453, N405	F456
	Zn/Zn	Q453	No Interactions
Compound **2** (*R*)	Zn/Mg	Q453, A452, N405	F456
	Mg/Mg	Q453, A452	F456
	Zn/Zn	Q453, A452	Y424
Compound **2** (*S*)	Zn/Mg	Q453, Y424	F456, H368
	Mg/Mg	Q453, A452	F456, H368
	Zn/Zn	Q453, A452	F456
Compound **3** (*R*)	Zn/Mg	T302	Y424, F441
	Mg/Mg	No Interactions	No Interactions
	Zn/Zn	Q453	F456
Compound **3** (*S*)	Zn/Mg	Q453, A452	F456
	Mg/Mg	Q453, A452, Y424	F456
	Zn/Zn	Y424, A452, F456	F456
Compound **4**	Zn/Mg	Q453	F456, F441
	Mg/Mg	No Interactions	F456
	Zn/Zn	Q453	F456
Compound **5**	Zn/Mg	No Interactions	No Interactions
	Mg/Mg	A452	F441
	Zn/Zn	A452	F456, F441
Compound **6**	Zn/Mg	Q453, A452	F456
	Mg/Mg	Q453, A452	F456
	Zn/Zn	Q453, A452	F456

## Data Availability

All data presented in the paper.

## Supplementary Materials

The following are available online at https://www.mdpi.com/article/10.3390/biom11050709/s1, Figure S1. C-alpha RMSD plot of PDE-9 (Mg/Mg, Zn/Mg, Zn/Zn) in complexed with Compound-1R; Figure S2. C-alpha RMSD plot of PDE-9 (Mg/Mg, Zn/Mg, Zn/Zn) in complexed with Compound-1S; Figure S3. C-alpha RMSD plot of PDE-9 (Mg/Mg, Zn/Mg, Zn/Zn) in complexed with Compound-2R; Figure S4. C-alpha RMSD plot of PDE-9 (Mg/Mg, Zn/Mg, Zn/Zn) in complexed with Compound-2S; Figure S5. C-alpha RMSD plot of PDE-9 (Mg/Mg, Zn/Mg, Zn/Zn) in complexed with Compound-3R; Figure S6. C-alpha RMSD plot of PDE-9 (Mg/Mg, Zn/Mg, Zn/Zn) in complexed with Compound-3S; Figure S7. C-alpha RMSD plot of PDE-9 (Mg/Mg, Zn/Mg, Zn/Zn) in complexed with Compound-4; Figure S8. C-alpha RMSD plot of PDE-9 (Mg/Mg, Zn/Mg, Zn/Zn) in complexed with Compound-5; Figure S9. C-alpha RMSD plot of PDE-9 (Mg/Mg, Zn/Mg, Zn/Zn) in complexed with Compound-6; Figure S10. Protein RMSF plot of PDE-9 (Mg/Mg, Zn/Mg, Zn/Zn) in complexed with Compound-6; Figure S11. Protein RMSF plot of PDE-9 (Mg/Mg, Zn/Mg, Zn/Zn) in complexed with Compound-6; Figure S12. Protein RMSF plot of PDE-9 (Mg/Mg, Zn/Mg, Zn/Zn) in complexed with Compound-6; Figure S13. Protein RMSF plot of PDE-9 (Mg/Mg, Zn/Mg, Zn/Zn) in complexed with Compound-6; Figure S14. Protein RMSF plot of PDE-9 (Mg/Mg, Zn/Mg, Zn/Zn) in complexed with Compound-6; Figure S15. Protein RMSF plot of PDE-9 (Mg/Mg, Zn/Mg, Zn/Zn) in complexed with Compound-6; Figure S16. Protein RMSF plot of PDE-9 (Mg/Mg, Zn/Mg, Zn/Zn) in complexed with Compound-6; Figure S17. Protein RMSF plot of PDE-9 (Mg/Mg, Zn/Mg, Zn/Zn) in complexed with Compound-6; Figure S18. Protein RMSF plot of PDE-9 (Mg/Mg, Zn/Mg, Zn/Zn) in complexed with Compound-6; Figure S19. Electrostatic interaction map using APBS calculation for the Compound-3S in all three different metal systems (Mg/Mg, Zn/Mg, Zn/Zn).; Figure S20. Electrostatic interaction map using APBS calculation for the Compound-6 in all three different metal systems (Mg/Mg, Zn/Mg, Zn/Zn).
