# Supplementary material for: Computational Study on Selective PDE9 Inhibitors on PDE9-Mg/Mg, PDE9-Zn/Mg, and PDE9-Zn/Zn Systems
Source: Biomolecules. 2021 May 10;11(5):709. doi: 10.3390/biom11050709 (PMC8151263; doi:10.3390/biom11050709)
Supplement: Supplementary file 1 [file biomolecules-11-00709-s001.zip › biomolecules-1195604-supplementary.pdf]

Supplementary Information

Computational Study on Selective PDE9 inhibitors on PDE9-Mg/Mg, PDE9-Zn/Mg, and PDE9-Zn/Zn systems

Dakshinamurthy Sivakumar[1], Sathish-Kumar Mudedla[1], Seonghun Jang[1], Hyunjun Kim[2], Hyunjin Park[2], Yong-won Choi[2], Joongyo Oh[2] and Sangwook Wu[1,*]

[1] R&D Center, Pharmcadd, 221, 17 APEC-ro, Haeundae-gu, Busan 48060, Republic of Korea
[2] R&D Center, Huons co. Ltd., Ansan-si, Gyeonggi-do, 15588, Republic of Korea
*Correspondence: s.wu@pharmcadd.com; Tel.: 82-51-731-5688

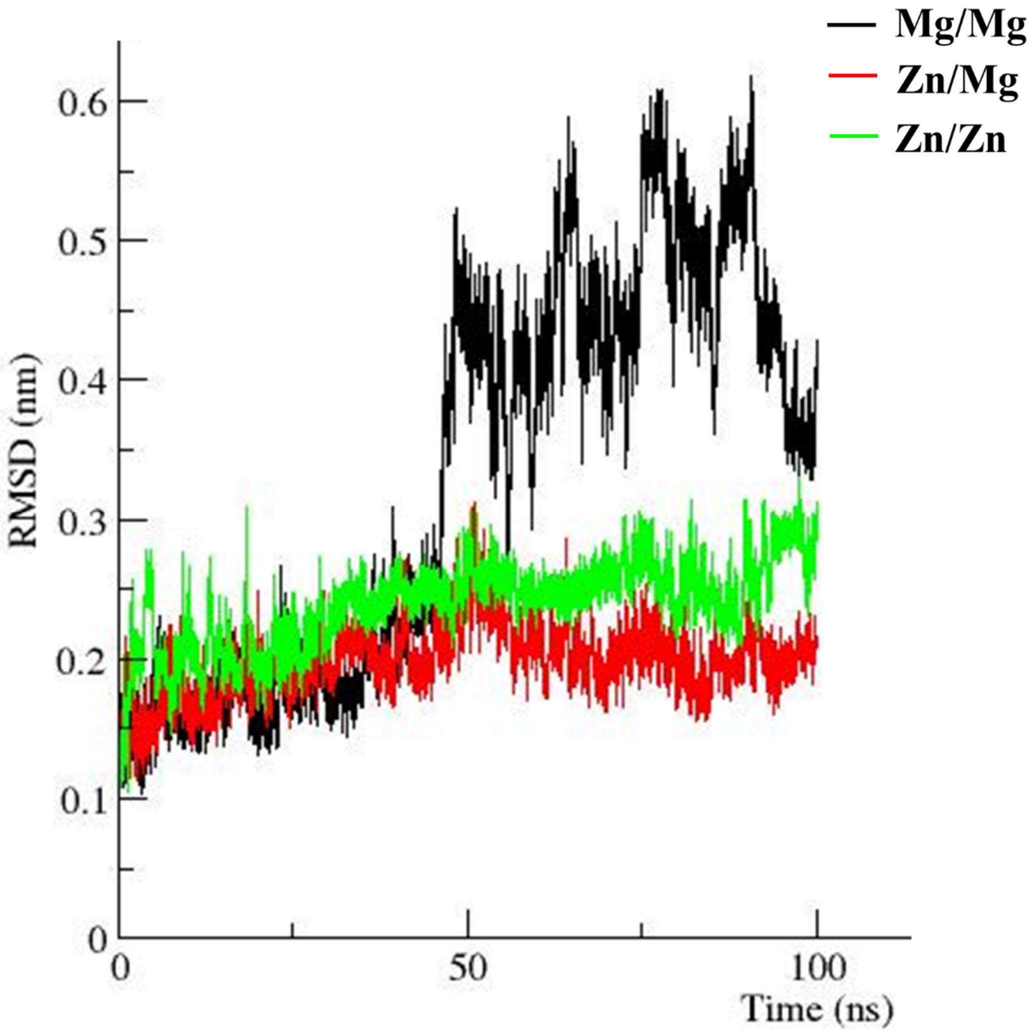

Figure

Figure S1. C-alpha RMSD plot of PDE-9 (Mg/Mg, Zn/Mg, Zn/Zn) in complexed with compound-1R.

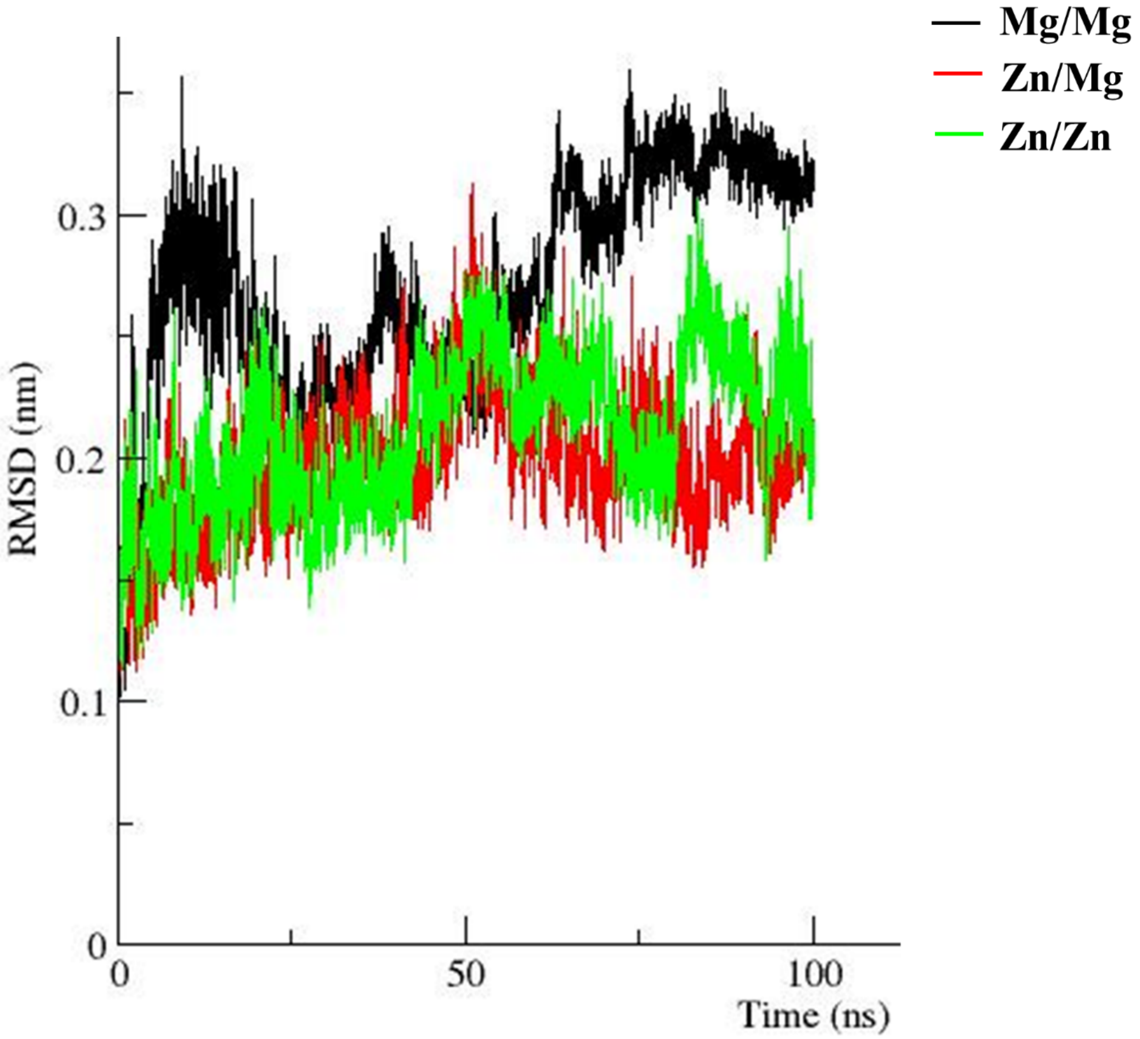

Figure S2. C-alpha RMSD plot of PDE-9 (Mg/Mg, Zn/Mg, Zn/Zn) in complexed with compound-1S.

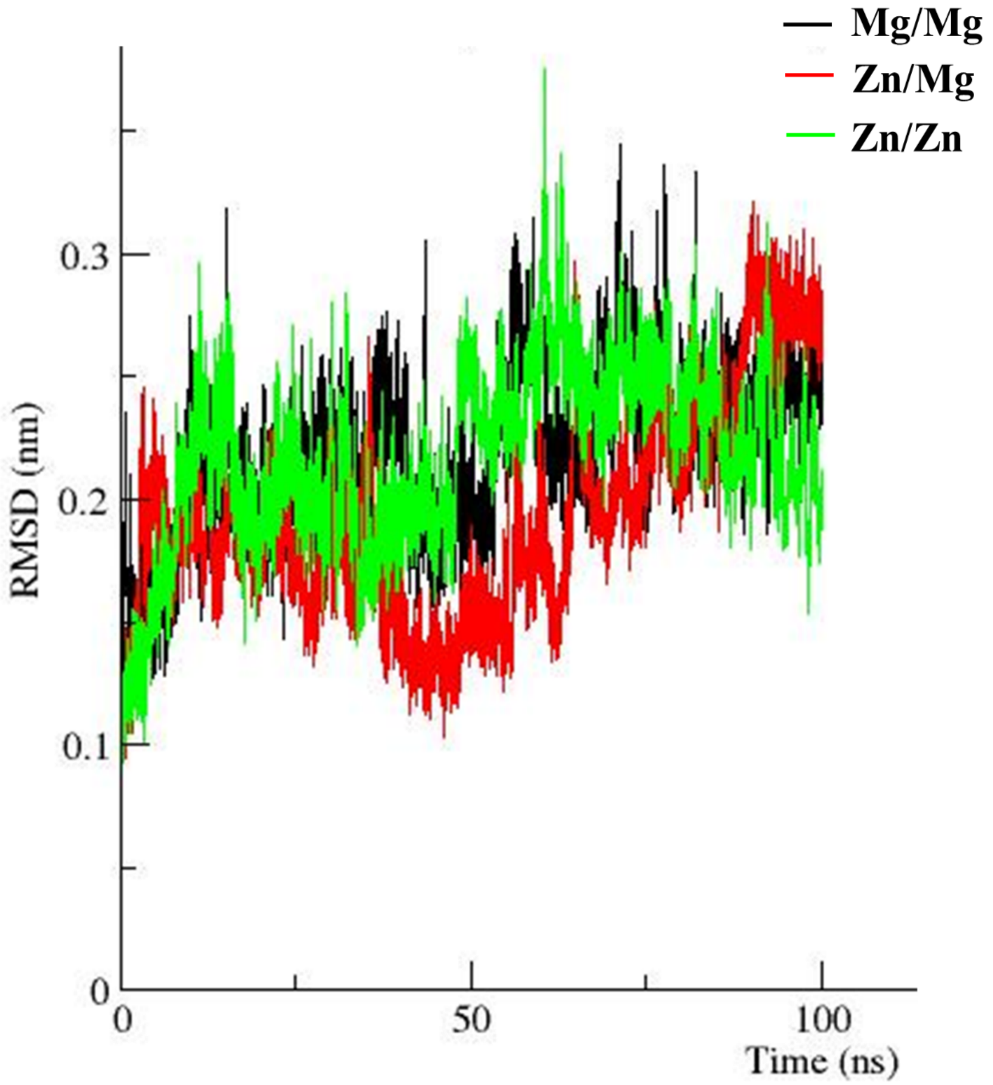

Figure S3.

Figure S3. C-alpha RMSD plot of PDE-9 (Mg/Mg, Zn/Mg, Zn/Zn) in complexed with compound-2R.

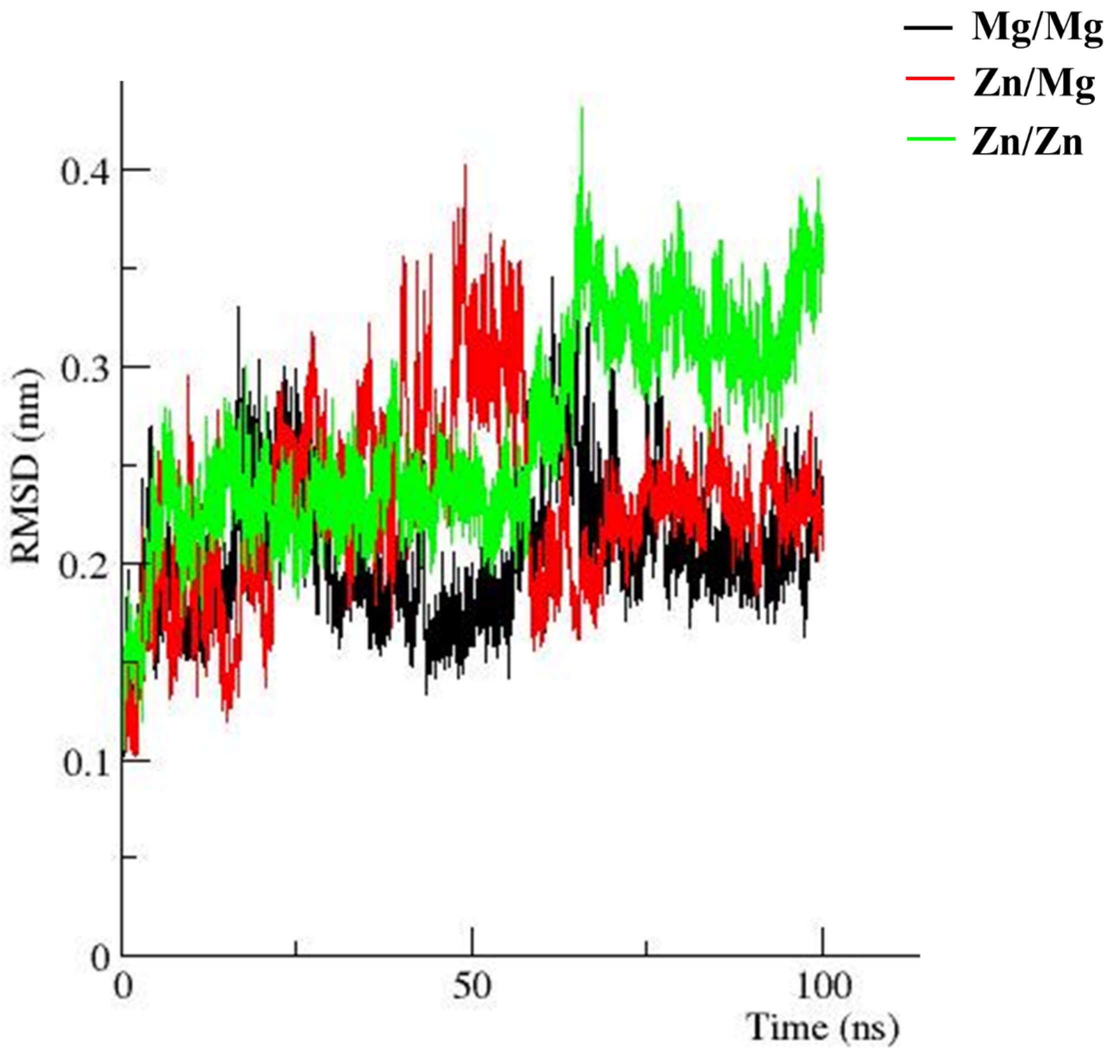

Figure S4. C-alpha RMSD plot of PDE-9 (Mg/Mg, Zn/Mg, Zn/Zn) in complexed with compound-2S.

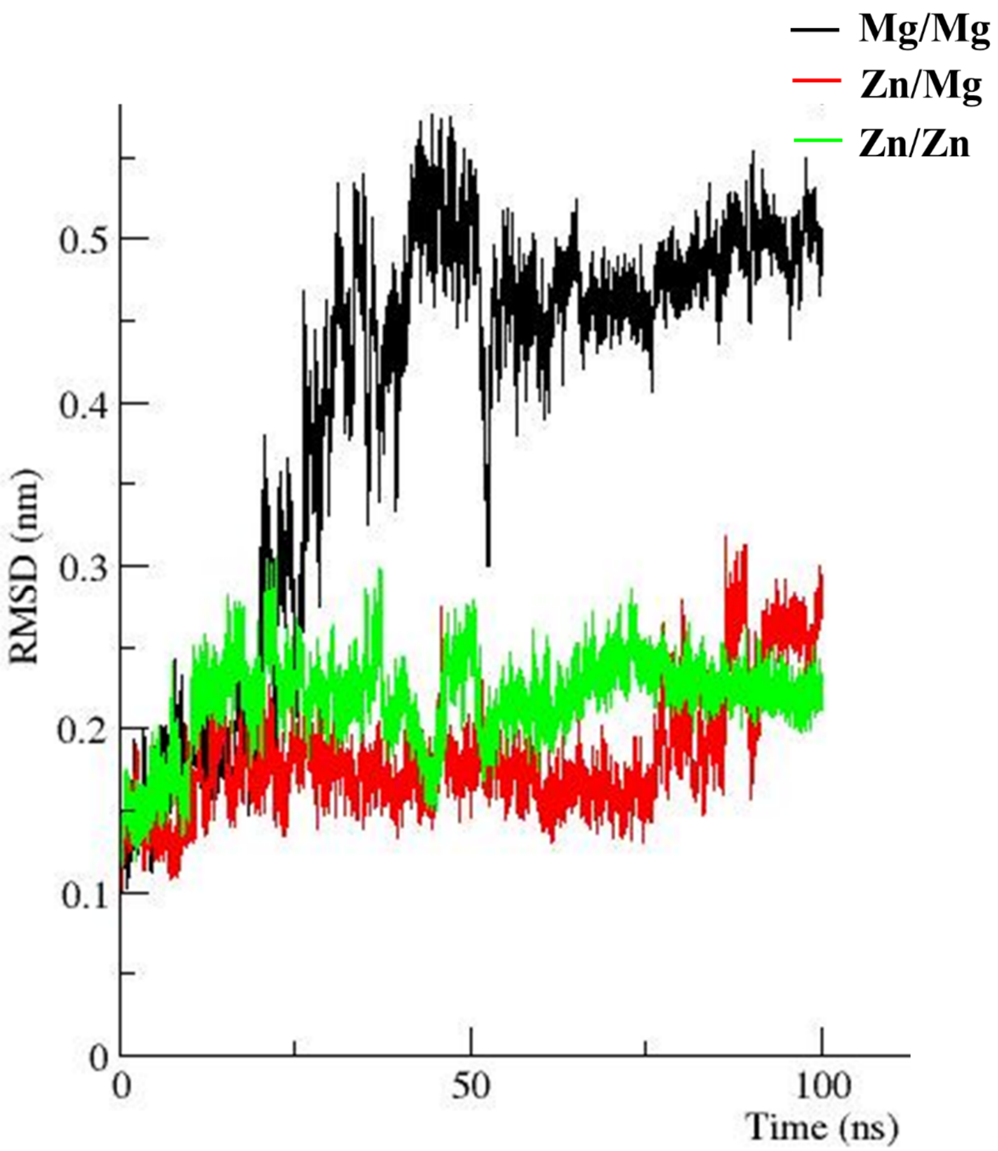

Figure S5.

Figure S5. C-alpha RMSD plot of PDE-9 (Mg/Mg, Zn/Mg, Zn/Zn) in complexed with compound-3R.

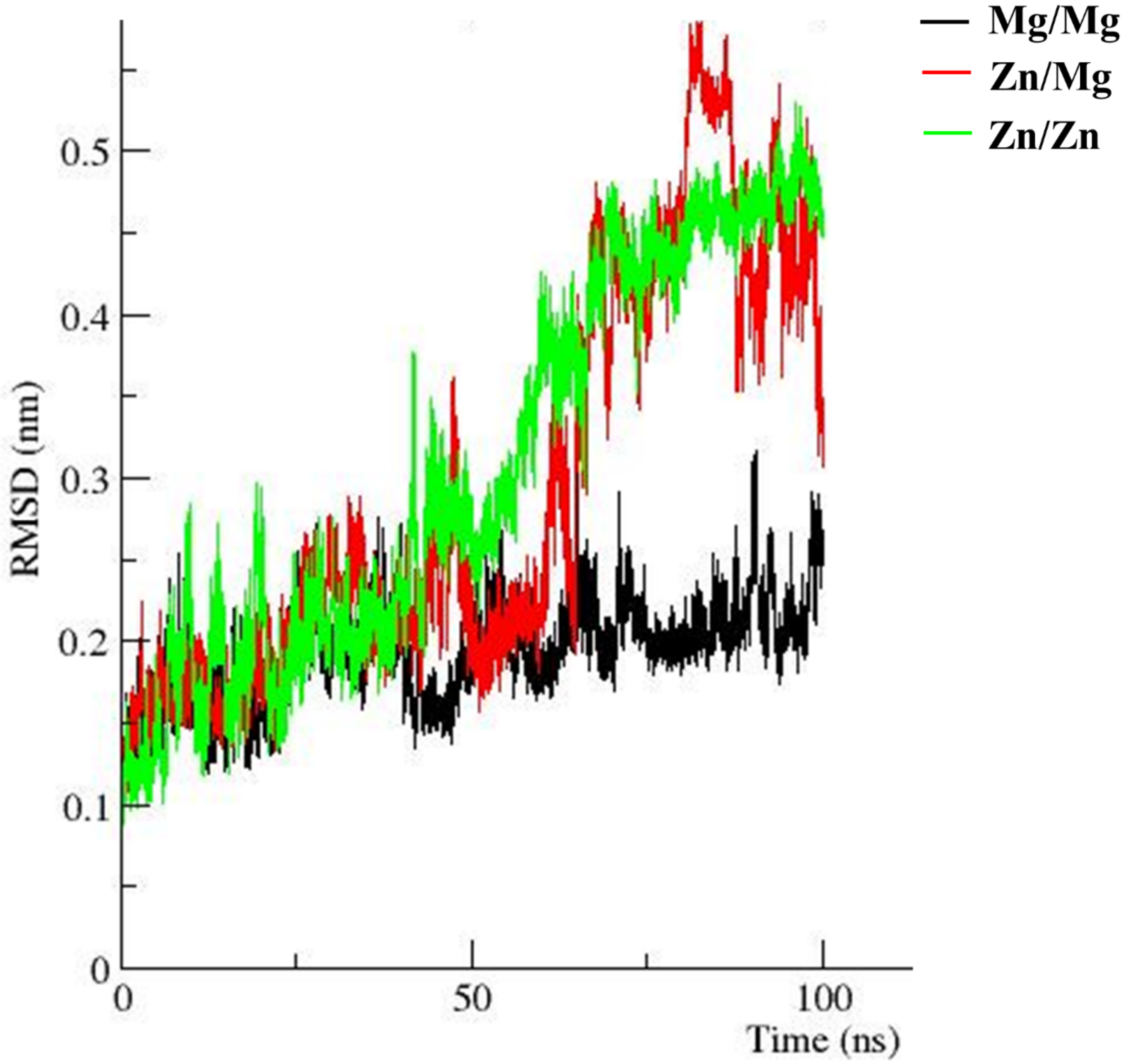

Figure S6. C-alpha RMSD plot of PDE-9 (Mg/Mg, Zn/Mg, Zn/Zn) in complexed with compound-3S.

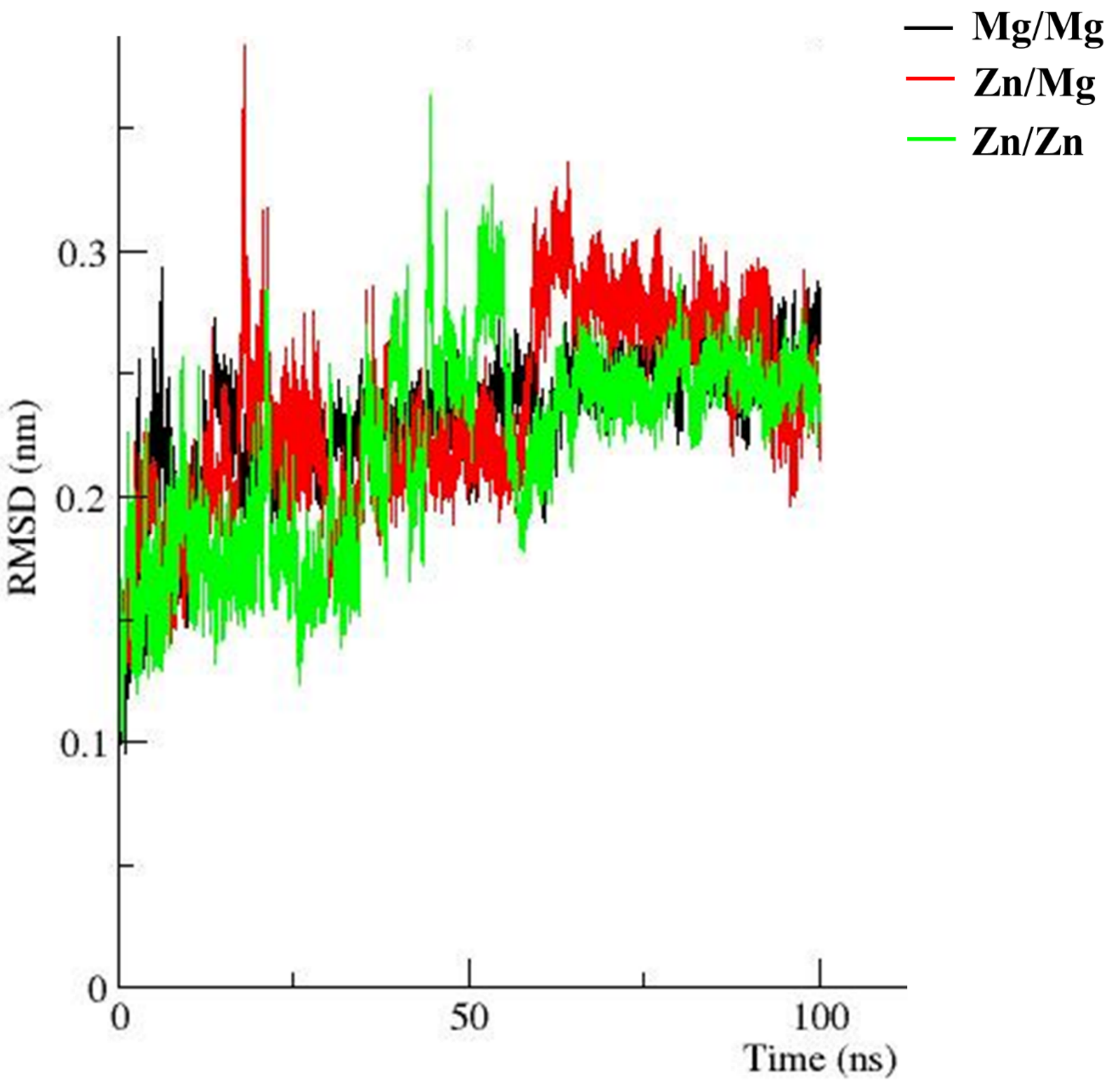

Figure S7. C-alpha RMSD plot of PDE-9 (Mg/Mg, Zn/Mg, Zn/Zn) in complexed with compound-4.

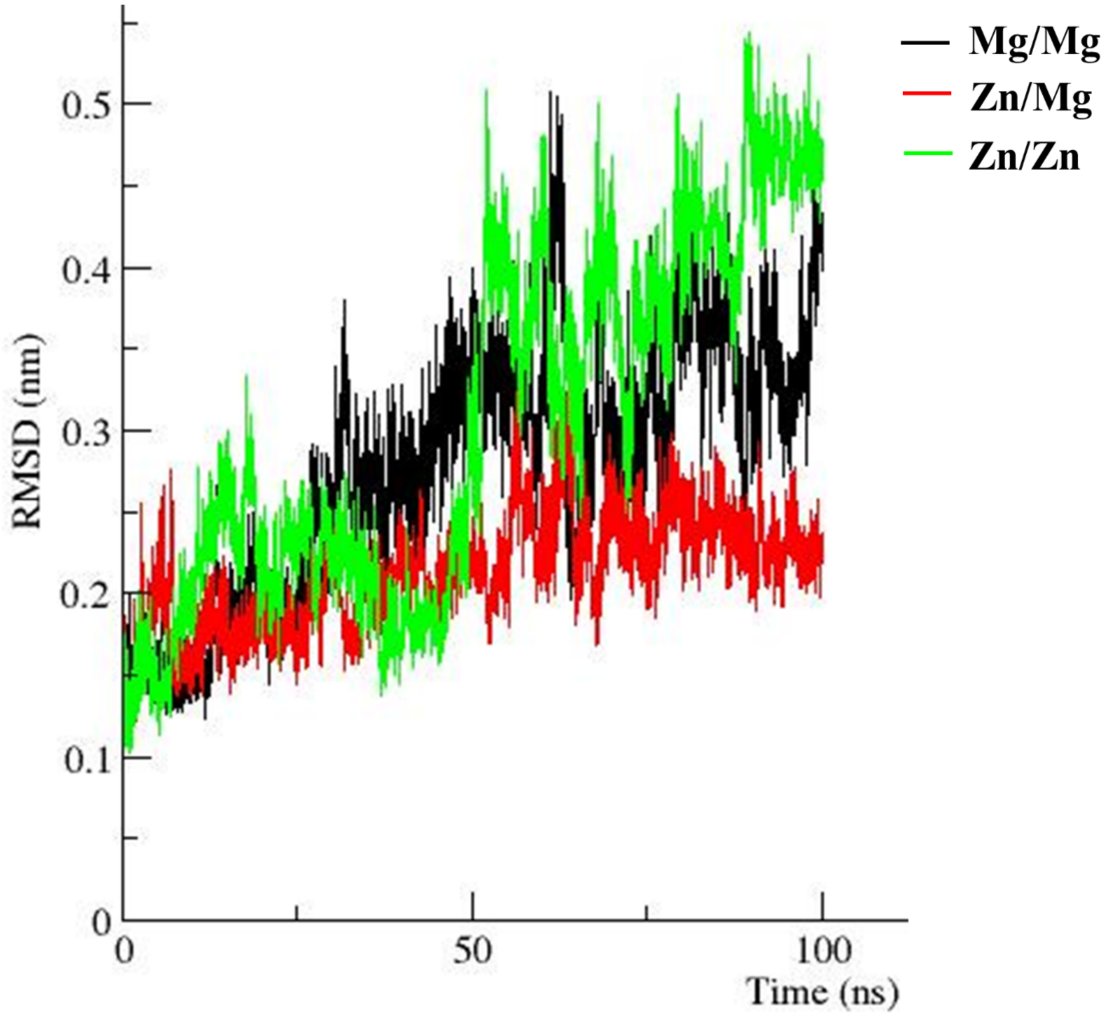

Figure S8. C-alpha RMSD plot of PDE-9 (Mg/Mg, Zn/Mg, Zn/Zn) in complexed with compound-5.

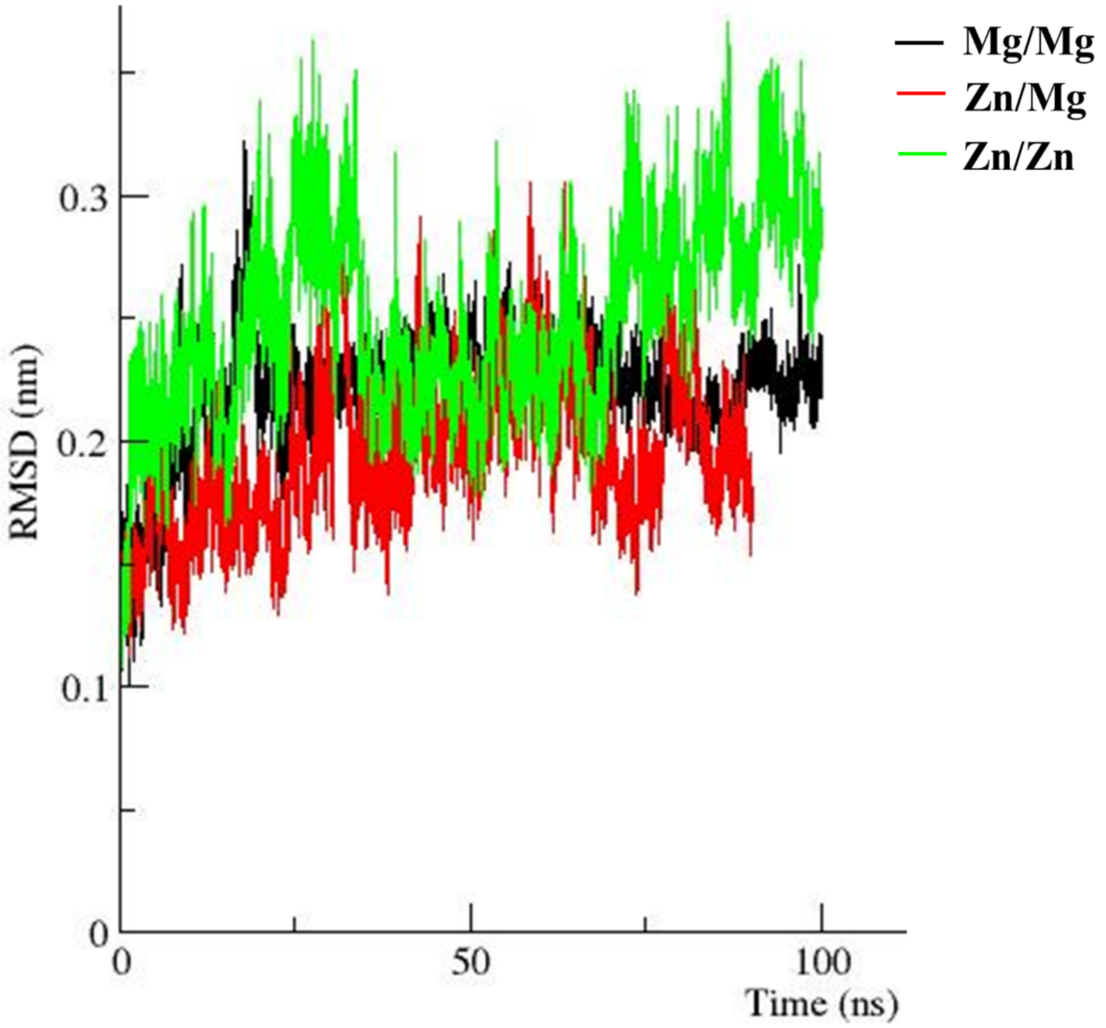

Figure S9. C-alpha RMSD plot of PDE-9 (Mg/Mg, Zn/Mg, Zn/Zn) in complexed with compound-6.

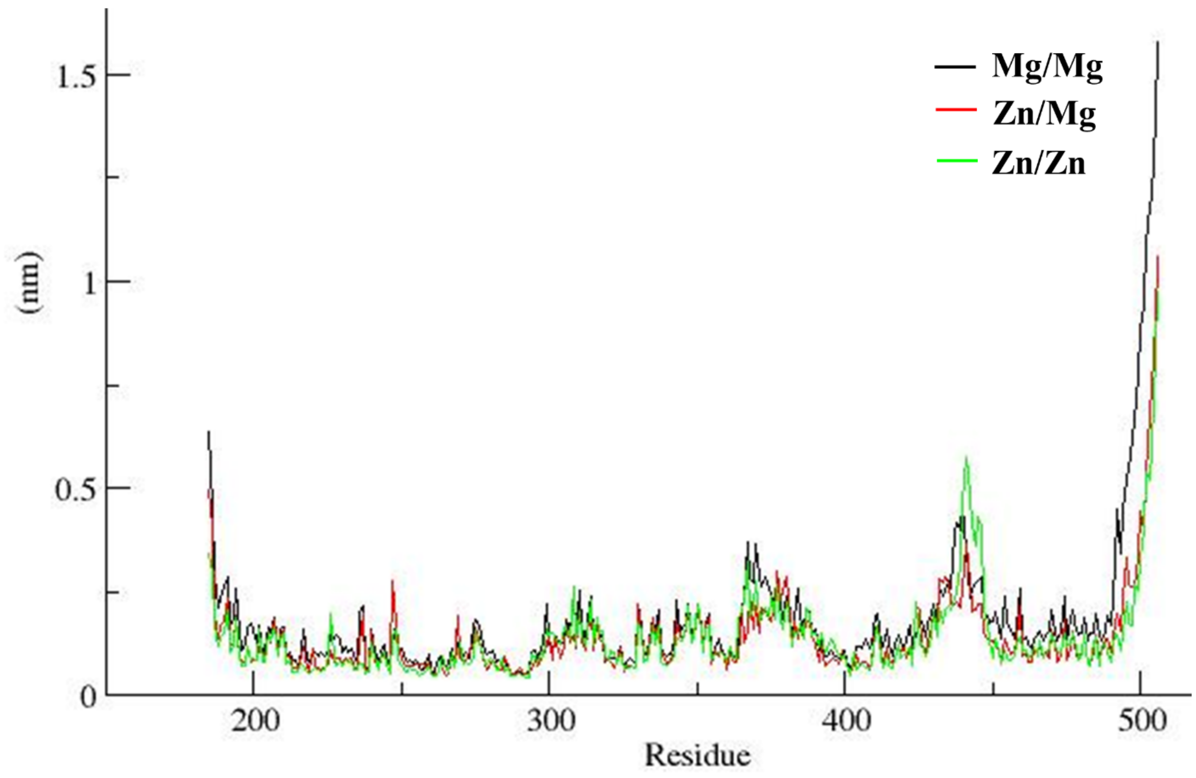

Figure S10. Protein RMSF plot of PDE-9 (Mg/Mg, Zn/Mg, Zn/Zn) in complexed with compound-6.

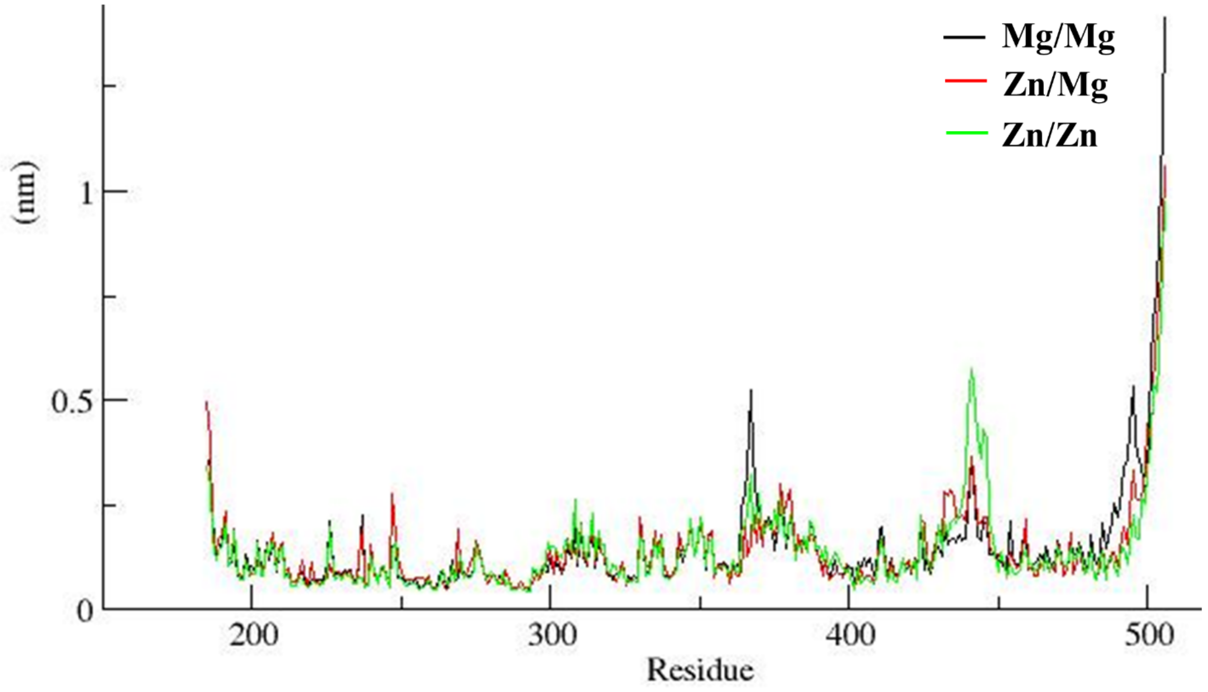

Figure S11. Protein RMSF plot of PDE-9 (Mg/Mg, Zn/Mg, Zn/Zn) in complexed with compound-6.

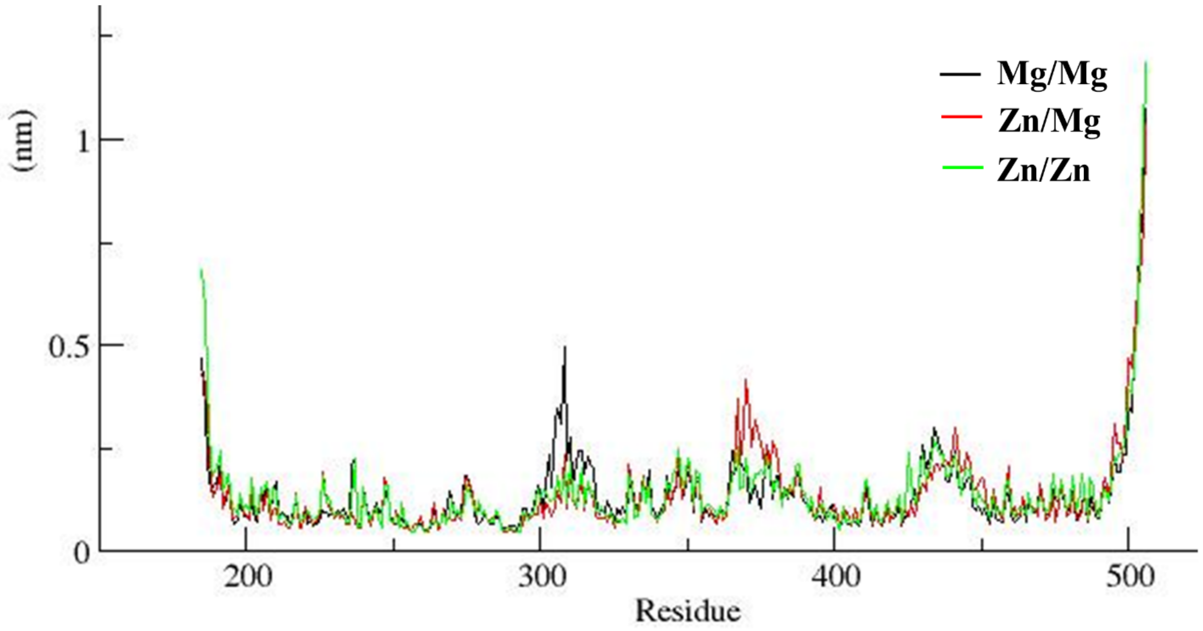

Figure S12. Protein RMSF plot of PDE-9 (Mg/Mg, Zn/Mg, Zn/Zn) in complexed with compound-6.

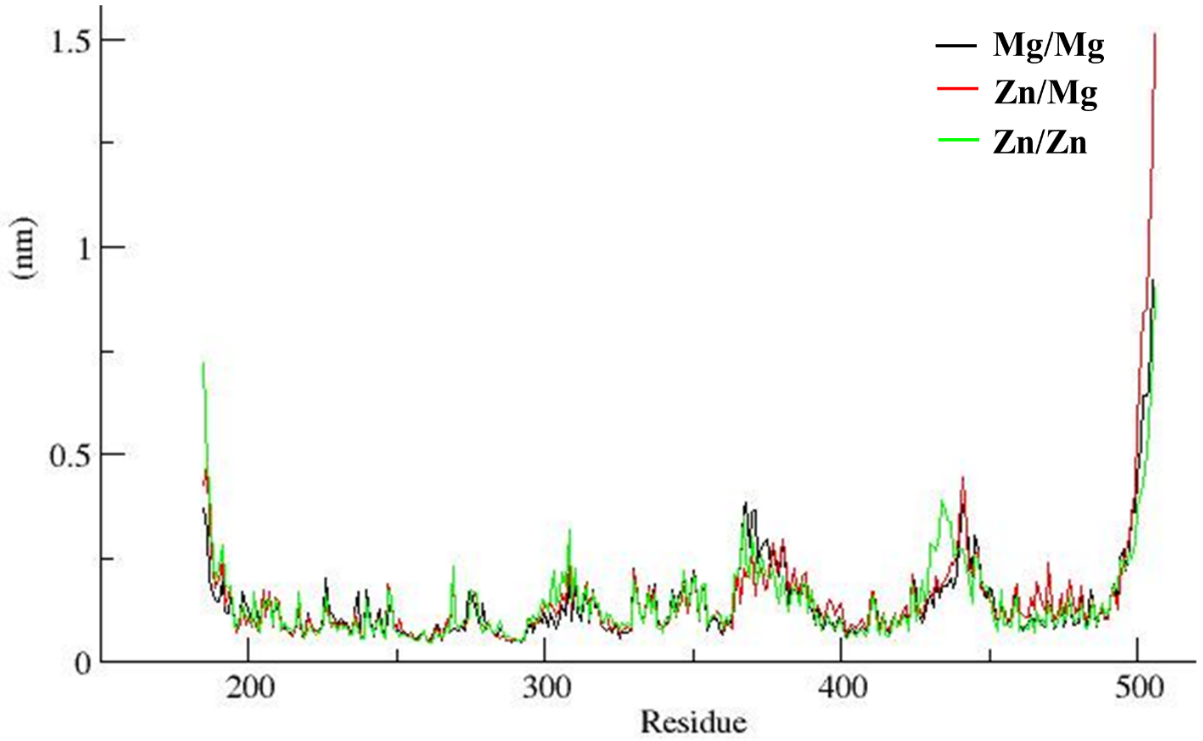

Figure S13. Protein RMSF plot of PDE-9 (Mg/Mg, Zn/Mg, Zn/Zn) in complexed with compound-6.

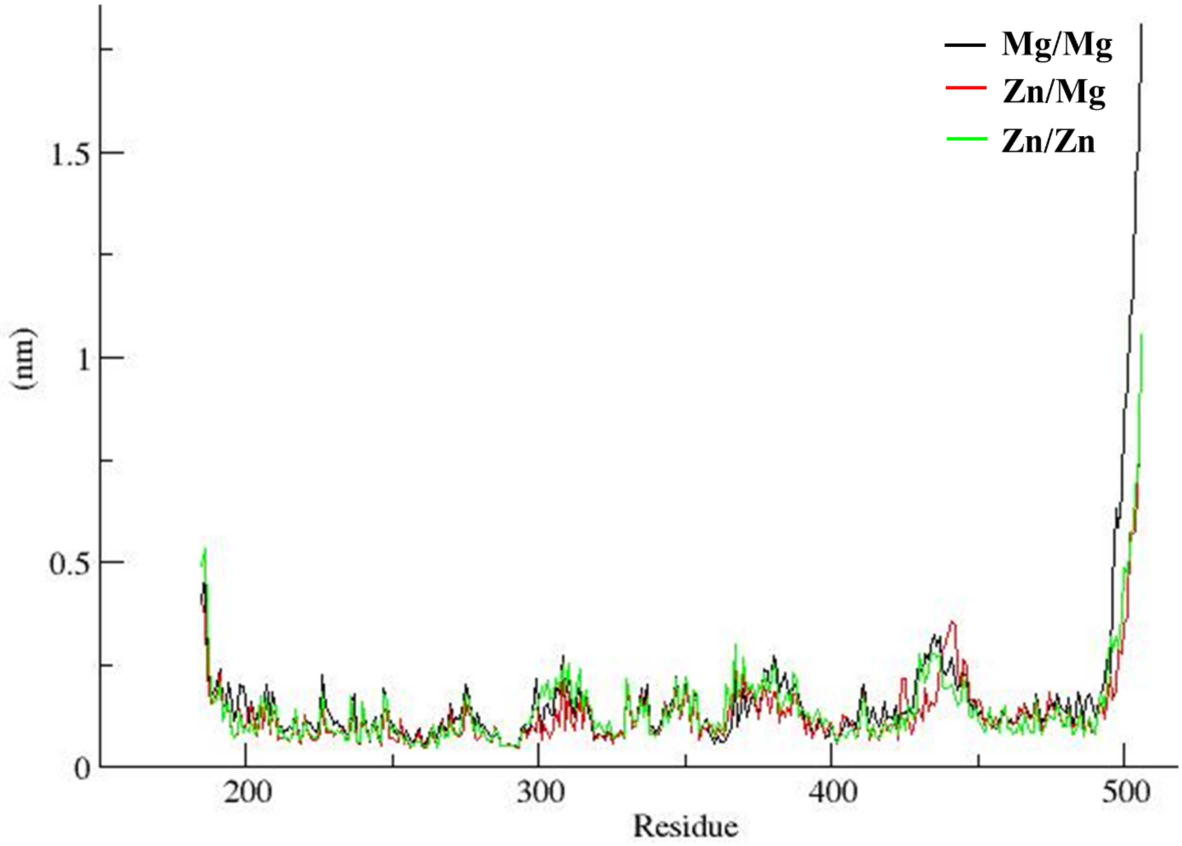

Figure S14. Protein RMSF plot of PDE-9 (Mg/Mg, Zn/Mg, Zn/Zn) in complexed with compound-6.

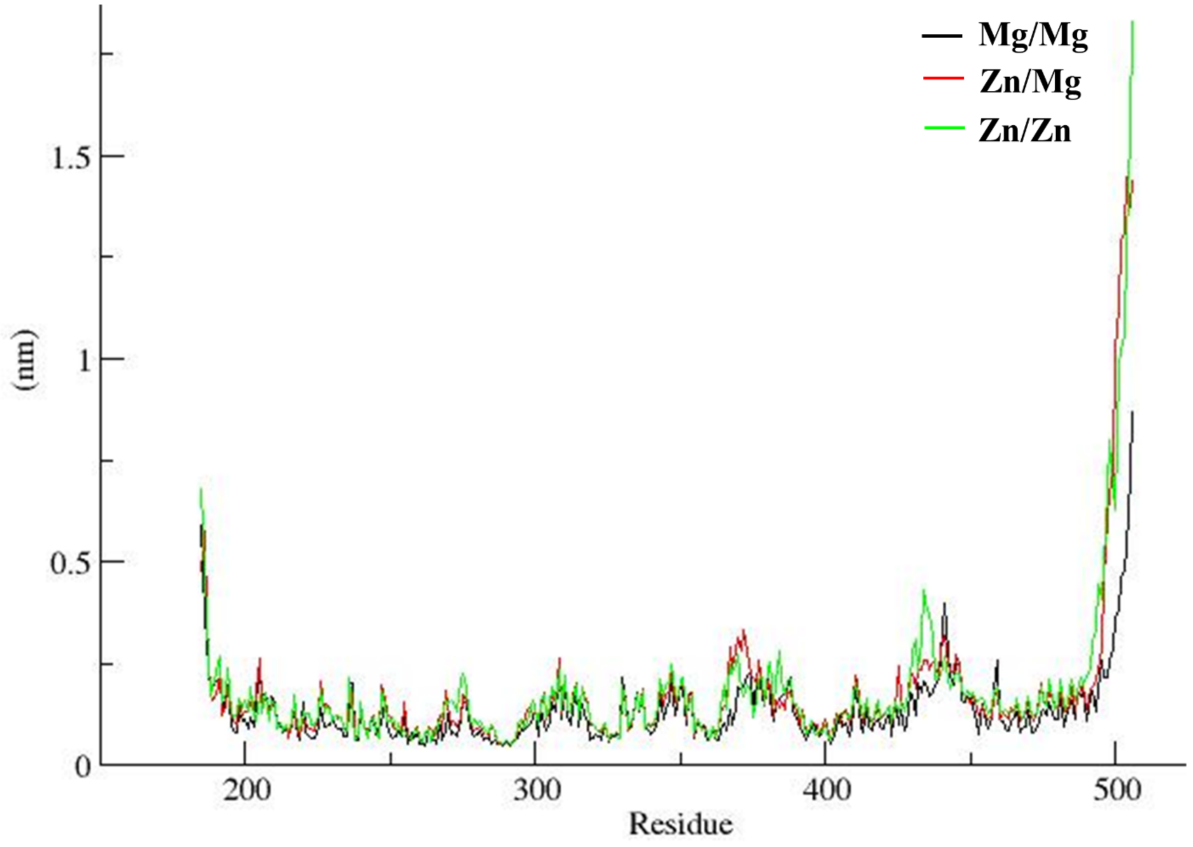

Figure S15. Protein RMSF plot of PDE-9 (Mg/Mg, Zn/Mg, Zn/Zn) in complexed with compound-6.

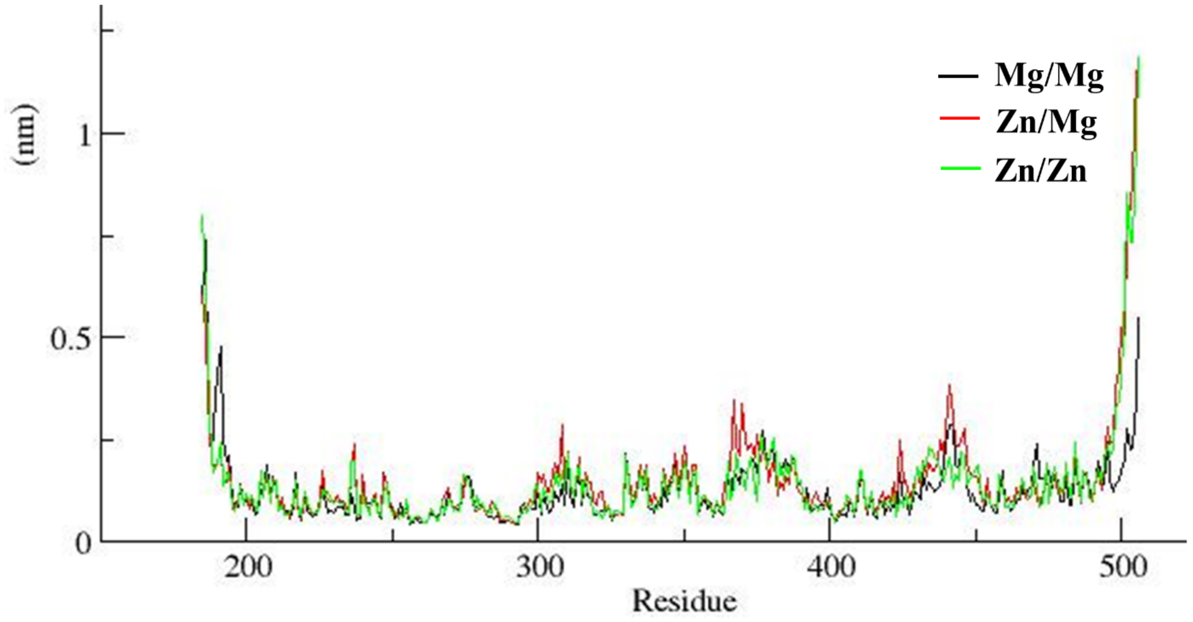

Figure S16. Protein RMSF plot of PDE-9 (Mg/Mg, Zn/Mg, Zn/Zn) in complexed with compound-6.

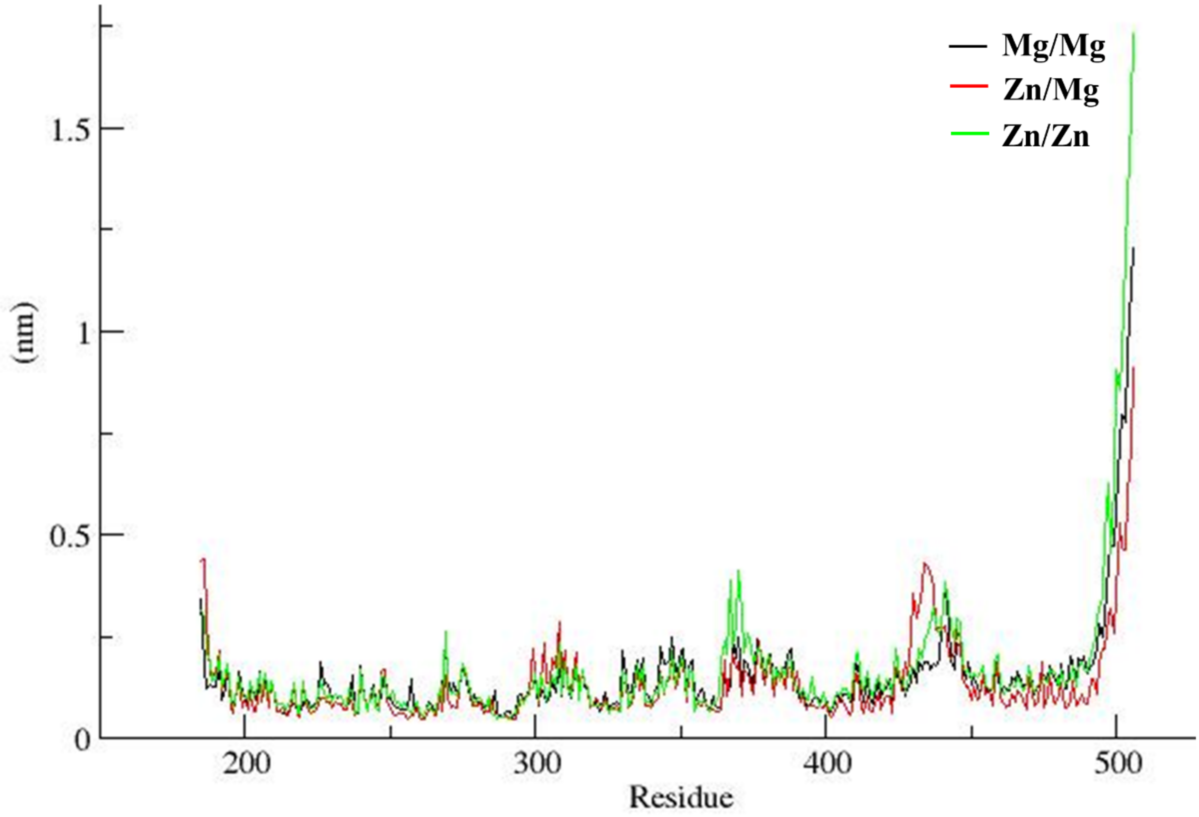

Figure S17. Protein RMSF plot of PDE-9 (Mg/Mg, Zn/Mg, Zn/Zn) in complexed with compound-6.

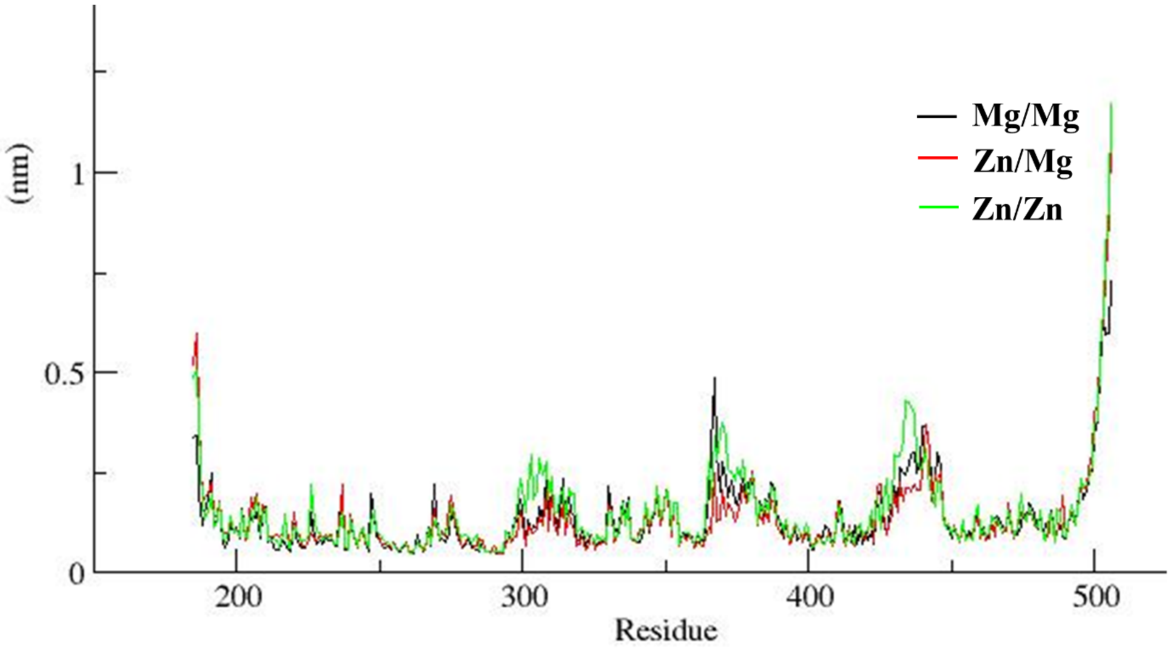

Figure S18. Protein RMSF plot of PDE-9 (Mg/Mg, Zn/Mg, Zn/Zn) in complexed with compound-6.

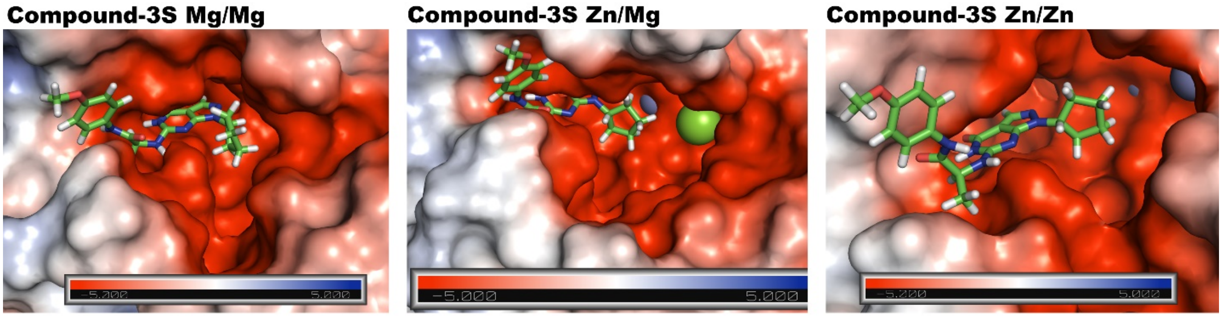

Figure S19. Electrostatic interaction map using APBS calculation for the Compound-3S in all three different metal systems (Mg/Mg, Zn/Mg, Zn/Zn).

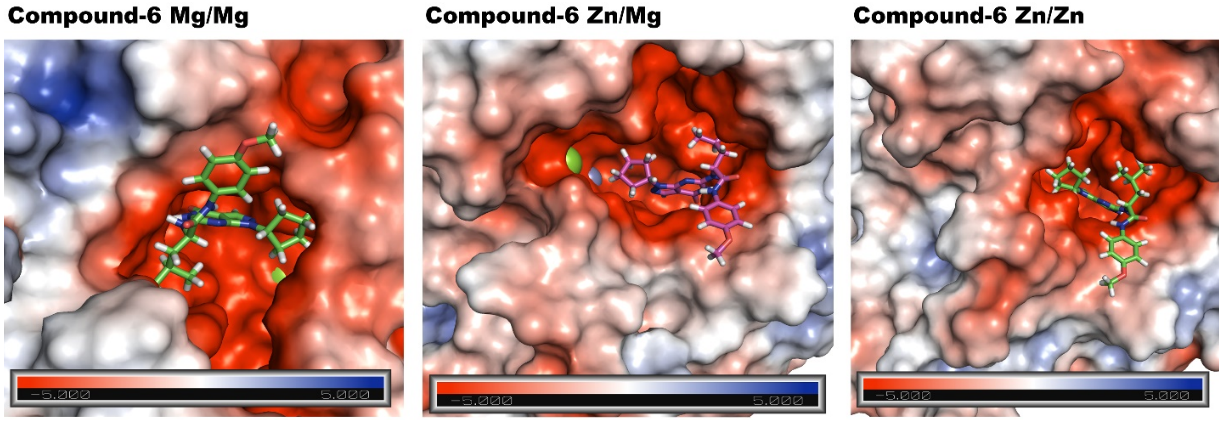

Figure S20. Electrostatic interaction map using APBS calculation for the Compound-6 in all three different metal systems (Mg/Mg, Zn/Mg, Zn/Zn).
